# The Role of Acetylcholinesterase Inhibitors in the Treatment of Prolonged Postelectroconvulsive Therapy Delirium

**DOI:** 10.1155/2022/6966882

**Published:** 2022-05-30

**Authors:** Brianna Gutowski, Emily Bomasang-Layno

**Affiliations:** Christiana Care Health System, 4755 Ogletown Stanton Rd, Newark, DE 19718, USA

## Abstract

Electroconvulsive therapy (ECT) is an extremely effective treatment modality for severe depression but is often associated with transient or persistent cognitive impairment. ECT-induced cognitive impairment, however, can serve as a deterrent to completion of treatment. We present a case of a prolonged post-ECT delirium lasting approximately 3 weeks in which donepezil, an acetylcholinesterase inhibitor, was used and was successful in shortening the duration of post-ECT delirium.

## 1. Introduction

Electroconvulsive therapy (ECT) is a safe and efficacious treatment modality that has been utilized since 1938 to treat several psychiatric conditions including severe depression, bipolar disorder, schizophrenia, and catatonia [1, 2]. ECT can produce minor adverse effects such as headache, myalgia, and nausea [3, 4]. However, one of the more concerning adverse effects often seen with ECT is cognitive impairment. ECT-induced cognitive impairment includes transient delirium, anterograde amnesia (i.e., difficulty retaining new information), and retrograde amnesia (i.e., impaired recall of memories formed prior to ECT) [3–5]. Post-ECT delirium can occur in up to 36% of patients receiving ECT [6–8]. In some cases, however, the delirium following ECT may be prolonged or even refractory to the typical modalities of treatment [8]. In cases of prolonged post-ECT delirium, the use of acetylcholinesterase inhibitors such as donepezil, rivastigmine, and galantamine may be beneficial in reducing the duration of post-ECT delirium [9].

## 2. Case Presentation

The patient is a 78-year-old Caucasian female with a past medical history of breast cancer in remission, hyperlipidemia, and hypothyroidism and a past psychiatric history of major depressive disorder, generalized anxiety disorder, and complicated grief following the passing of her husband of 55 years 24 months prior to presentation. The patient was admitted to a medical psychiatry unit for evaluation and treatment of severe depression characterized by poor self-care, anhedonia, fragmented sleep, early morning awakening, amotivation, impaired concentration, and an unintentional weight loss of approximately 40 pounds since her cancer diagnosis in 2016. The patient also endorsed soft auditory hallucinations described as music intermittently playing in her head. Her depression was accompanied by passive suicidal ideation as well as cognitive impairment, which was highly suspicious for the dementia syndrome of depression. Montreal Cognitive Assessment (MOCA) score was 15/30 with deficits in visuospatial/executive functioning, naming, attention, and recall, although there was concern for poor concentration and effort. The patient also had multiple medication trials including escitalopram, sertraline, fluoxetine, quetiapine, and gabapentin. At the time of admission, she was on the following regimen: fluoxetine 20 mg daily, aripiprazole 6 mg daily, and lorazepam 0.25 mg daily at noon and at bedtime. The admitting diagnosis was major depressive disorder, single episode, severe, with psychotic features, and based on her clinical presentation, the treatment team strongly considered the initiation of an index course of ECT. In the interim, medication adjustments were made, namely, the cross titration of fluoxetine with duloxetine.

A pre-ECT medical assessment including complete blood count, serum electrolytes, liver and thyroid function tests, blood glucose, and electrocardiogram were all within normal limits. A recent head computed tomography (CT) scan was reviewed and was negative for acute intracranial abnormalities. An electroencephalogram (EEG) was not performed.

Right unilateral brief pulse (0.5 milliseconds) ECT was initiated using a Thymatron device thrice weekly with a stimulus intensity of 20%. Methohexital 60 mg was used for anesthesia, and succinylcholine 50 mg was used as a muscle relaxant. During ECT, the following psychotropic medications were continued: duloxetine 30 mg twice daily and aripiprazole 6 mg daily. The patient received a total of six ECT treatments (summarized in Table 1). Following ECT treatments 2, 4, and 5, the patient experienced treatment emergent confusion, which resolved a few hours after treatment. This was characterized by disorientation, primarily with elements of place or time, requiring prompting or cueing with activities of daily living (ADLs). Some confabulation after treatment 5 was noted. Bouts of confusion were also more prominent in the evening. Following ECT treatment 6, the patient was noted to be confused and disoriented again. The patient intermittently endorsed visual hallucinations, had poor concentration, and was noted to be confabulating. Symptoms followed the typical waxing and waning pattern. This persisted for several days, prompting eventual discontinuation of further treatments. A repeat MOCA was performed 6 days after ECT treatment 6, and the patient scored 9/30 with deficits in visuospatial/executive functioning, attention, language, abstraction, recall, and orientation. Due to ongoing confusion, several psychotropic medications were tapered and eventually discontinued including aripiprazole. Duloxetine was maintained, and the patient was also started on low-dose quetiapine, 25 mg every morning and 50 mg at bedtime for management of delirium. Delirium precautions were maintained.

A thorough medical workup was performed to assess for underlying etiologies of a prolonged delirium. This work up included complete blood count (CBC), serum electrolytes, liver function tests, urinalyses, and head CT. CBC was unremarkable for a leukocytosis making infection as a potential etiology of her delirium unlikely. In addition, several urinalyses were negative for a urinary tract infection (UTI). There was no evidence of any electrolyte abnormalities or hepatic or renal impairment to precipitate a delirium. Head CT was able to rule out other potential CNS causes of delirium including stroke, hemorrhage, or neoplasm. The patient was also evaluated by neurology. Based on clinical assessment, the etiology of her delirium was determined to be a prolonged post-ECT delirium given the temporal relationship of the development of symptoms relative to treatment with ECT. Additionally, an underlying dementia at baseline was suspected, worsening in the setting of depression. The bouts of confusion persisted although some periods of lucidity were noted. On day 16 following the last ECT treatment, a trial of donepezil was started at 5 mg once daily in an attempt to shorten this bout of delirium.

Within four days, the patient's delirium began to clear, and she was observed to be more alert with minimal confusion. She required less cueing and less assistance with her ADLs. Orientation was also much more consistent and by day 8 after initiation of donepezil, she was noted to be very close to her baseline. Patient was also able to participate more meaningfully in treatment. Her final medication regimen consisted of duloxetine 30 mg twice daily, quetiapine 25 mg every morning and 50 mg at bedtime, and donepezil 5 mg every morning. With this medication regimen, in addition to the 6 ECT treatments she received, her mood also improved, and the passive suicidal ideation had cleared.

## 3. Discussion

ECT is a highly effective and generally well-tolerated treatment modality for depression. Associated cognitive impairment, however, can at times serve as a deterrent to initiate treatment or may result in early termination of treatment. ECT postictal delirium is one of the distinct cognitive side effects of ECT with variable incidence rates ranging from 5 to 36% [10–13]. It can present as agitated delirium on emergence from anesthesia [14]. More commonly observed is acute postictal disorientation immediately following treatment. Recovery from this is typically within 1 to 2 hours [15]. A third distinct presentation is interictal delirium, which is characterized by prolonged periods of disorientation immediately post-ECT and is separate from acute postictal disorientation. In addition to the prolonged confusion, features of hypomania have been described in adults over age 75 and have been noted to spontaneously resolve within 2 weeks of cessation of treatment [16]. Risk factors associated with postictal delirium include the presence of concomitant catatonia; presence of cerebrovascular disease, Parkinson's disease (PD), or dementia; bitemporal stimulation; high stimulus intensity; longer seizure length; use of dexmedetomidine or etomidate; higher body mass index (BMI); concomitant use of high-dose quetiapine; and the lack of use of antidepressants [10, 11, 13, 17, 18]. Age has been described as a risk factor for acute postictal disorientation, more specifically time to reorientation [19].

There are no pharmacologic guidelines currently available for ECT-induced cognitive impairment [20]. Different interventions have been investigated to potentially prevent postictal delirium, including the use of ultrabrief pulse ECT, low-dose dexmedetomidine pretreatment, olanzapine, and diazepam/haloperidol [17, 21–23]. Acetylcholinesterase inhibitors have been investigated primarily to attenuate ECT-induced cognitive impairment and have shown promising results [20, 24–26]. The cholinergic system is thought to play a particularly significant role in memory, learning, and attention. This same pathway and the glutamate pathway have both been implicated in cognitive impairment post-ECT. Acetylcholinesterase activity is thought to increase with electroconvulsive stimulation [20, 27]. Acetylcholinesterase inhibitors have therefore been investigated to potentially mitigate cognitive impairment or even shorten postictal delirium. Several case reports of post-ECT delirium have described successful treatment with the addition of donepezil [8, 28, 29]. The use of transdermal rivastigmine has also been described [5]. In these case reports, the acetylcholinesterase inhibitor was typically given either during active treatment, to address acute postictal disorientation, or a few days after ECT for postictal delirium. Of note, all cases showed a very good response with resolution of confusion within a few days of initiation of treatment. No side effects were reported as well.

Our case demonstrates bouts of acute postictal disorientation that quickly resolved and prolonged interictal delirium following the sixth ECT. Right unilateral ECT was utilized to try to mitigate any anticipated bouts of confusion. Possible risk factors for the development of prolonged delirium following ECT include a possible underlying neurocognitive disorder and this patient's age. Clinical presentation followed a waxing and waning course. There were also more apparent symptoms in the late afternoon into the evening, similar to the sundowning phenomenon seen in dementia. The expectation was for the delirium to resolve following cessation of treatment. However, symptoms persisted for 16 days. With the eventual initiation of low-dose donepezil (5 mg), there was a notable improvement in disorientation and clearing of perceptual disturbances, and prompting for the patient's ADLs was no longer required. The patient also became more engaged and was able to increase participation in unit activities. The patient was close to baseline after 4 days on donepezil. No adverse effects were observed or reported.

## 4. Conclusion

This case illustrates a very prolonged post-ECT delirium which showed minimal response to the usual interventions for delirium, including cessation of treatment, delirium precautions, the use of low-dose antipsychotics, and streamlining of the medication regimen. The positive response to donepezil, despite its late initiation, shows that there is benefit to its use regardless of timing. In retrospect, anticipatory administration of an acetylcholinesterase inhibitor could have been helpful as the patient was already experiencing postictal disorientation after most of her treatments. The utility of acetylcholinesterase inhibitors for postictal delirium is distinctly shown in this case. With the paucity of pharmacologic recommendations for treatment, larger controlled trials evaluating this drug class would be very valuable.

## Figures and Tables

**Table 1:** Summary of ECT treatments and response.

ECT treatment #	Anesthetic	Laterality	Stimulus intensity	Charge	Tonic-clonic seizure duration	EEG seizure duration	Acute confusion state (yes/no)
1	Methohexital/succinylcholine	Right unilateral	Low 0.5	20%	22 sec	49 sec	No
2	Methohexital/succinylcholine	Right unilateral	Low 0.5	20%	11 sec	38 sec	Yes: transient treatment emergent confusion, quickly resolved
3	Methohexital/succinylcholine	Right unilateral	Low 0.5	20%	19 sec	42 sec	No
4	Methohexital/succinylcholine	Right unilateral	Low 0.5	20%	14 sec	46 sec	Yes: disorientation emerging from anesthesia
5	Methohexital/succinylcholine	Right unilateral	Low 0.5	20%	15 sec	40 sec	Yes: increased disorientation emerging from anesthesia
6	Methohexital/succinylcholine	Right unilateral	Low 0.5	20%	14 sec	43 sec	Yes: prolonged disorientation began

## Data Availability

The observational and experimental data used to support this case report are cited at relevant places within the text as references [1–29].
